# Mechanisms, Management and Prognosis of Paraneoplastic Hypercalcemia in Penile Squamous Cell Carcinoma: A Structured Review

**DOI:** 10.3390/jcm15051809

**Published:** 2026-02-27

**Authors:** Andrei Andreșanu, Constantin Gîngu, Mihaela Roxana Oliță, Mihai Adrian Dobra, Bogdan Marian Sorohan, Bogdan Obrișcă, Dragoș Eugen Georgescu, Mihai Adrian Eftimie, Ioanel Sinescu

**Affiliations:** 1 Department of Urology, “Carol Davila” University of Medicine and Pharmacy, 020021 Bucharest, Romania; andrei.andresanu@drd.umfcd.ro (A.A.); constantin.gingu@umfcd.ro (C.G.); mihai.dobra@umfcd.ro (M.A.D.); ioanel.sinescu@umfcd.ro (I.S.); 2 Center of Uronephrology and Kidney Transplantation, Fundeni Clinical Institute, 022328 Bucharest, Romania; bogdan.sorohan@umfcd.ro (B.M.S.); bogdan.obrisca@umfcd.ro (B.O.); 3 Department of Anesthesiology and Intensive Care, “Carol Davila” University of Medicine and Pharmacy, 020021 Bucharest, Romania; 4 Anesthesiology and Intensive Care, Fundeni Clinical Institute, 022328 Bucharest, Romania; 5 Department of Nephrology, “Carol Davila” University of Medicine and Pharmacy, 020021 Bucharest, Romania; 6 Department of Surgery, “Carol Davila” University of Medicine and Pharmacy, 020021 Bucharest, Romania; dragos-eugen.georgescu@umfcd.ro (D.E.G.); mihai.eftimie@umfcd.ro (M.A.E.); 7 Department of General Surgery “I. Juvara”, “Dr. I. Cantacuzino” Clinical Hospital, 020471 Bucharest, Romania; 8 Department of Surgery, Fundeni Clinical Institute, 022328 Bucharest, Romania

**Keywords:** penile cancer, squamous cell carcinoma, paraneoplastic syndrome, hypercalcemia, PTHrP, bisphosphonates

## Abstract

**Background and Objectives:** Paraneoplastic hypercalcemia represents a rare but clinically significant complication of penile squamous cell carcinoma (PSCC); however, limited information is available for this condition. Therefore, this systematically conducted narrative review aimed to comprehensively evaluate the pathophysiological mechanisms, clinical presentation, therapeutic strategies and prognostic outcomes of tumor-induced hypercalcemia in PSCC. **Methods:** A comprehensive literature search was conducted across PubMed/MEDLINE and Scopus databases from their inception to December 2024. Cases were included if they documented histopathologically confirmed PSCC with biochemically verified hypercalcemia and objective evidence of paraneoplastic etiology. Data extraction included tumor characteristics, severity of hypercalcemia, mechanistic classification, therapeutic interventions and survival outcomes. The search methodology followed PRISMA 2020 guidelines adapted for narrative synthesis. Given the absence of comparative studies for this rare condition, all study types were eligible for inclusion, resulting in an evidence base that consisted exclusively of case reports and case series. **Results:** Twelve published cases spanning six decades (1965–2024) met the inclusion criteria. The median age at presentation was 56 years, with 91.6% of patients presenting with advanced disease. Severe hypercalcemia (≥14 mg/dL) occurred in 66.7% of cases, with a median calcium level of 15.45 mg/dL. Two established pathophysiological mechanisms were identified: PTHrP-mediated humoral hypercalcemia and bone metastasis-associated hypercalcemia. By contrast, three cases with unmeasured PTHrP levels had an undetermined mechanism. Despite biochemical correction, median overall survival was 9 weeks following diagnosis of hypercalcemia. **Conclusions:** Paraneoplastic hypercalcemia in PSCC represents a rare metabolic emergency. While aggressive management can achieve biochemical correction, the occurrence of hypercalcemia uniformly indicates advanced tumor biology with limited survival benefit. Early recognition and prompt multidisciplinary intervention remain essential for symptomatic relief and preserving quality of life. Reporting future cases and collaborating with international registries will be necessary to improve understanding of this rare paraneoplastic entity.

## 1. Introduction

Penile cancer (PC) represents a rare malignancy, with incidence rates ranging from 0.86 to 1.3 per 100,000 population in Central and Eastern European countries and an estimated mortality rate of 0.4 per 100,000. This disease disproportionately affects populations with low socio-economic status, reflecting limited access to preventive health measures and high prevalence of human papillomavirus (HPV) infection (particularly types 16 and 18). Despite known associations with risk factors such as phimosis, chronic inflammatory conditions (lichen sclerosus—LS), or tobacco use, the molecular pathogenesis remains incompletely characterized, and late-stage presentation continues to dominate in resource-limited settings [1].

Although localized penile squamous cell carcinoma (PSCC) is feasible for surgical treatment and demonstrates a favorable response, progression of this disease to advanced stages is associated with significantly poor clinical outcomes. Genitourinary tumors can cause paraneoplastic syndrome, even though renal cell carcinoma is the most frequent urological malignancy involved. Although some paraneoplastic complications, such as polycythemia and hypertension, are more typical of renal tumors, others, such as hypercalcemia and Cushing’s syndrome, are more commonly associated with other genitourinary cancers [2]. From this category of neoplasms, paraneoplastic syndromes are uncommonly encountered and reported in PSCC.

Tumor-induced hypercalcemia is predominantly observed in squamous cell carcinomas of the pulmonary, head and neck, and esophageal regions, with pathogenesis primarily attributed to humoral factors, notably the parathyroid hormone-related peptide (PTHrP) [3,4]. This condition has been documented in association with a range of genitourinary tumors. Among these, prostate cancer and renal cell carcinoma (RCC) are frequently noted [5,6]. This syndrome may manifest even in localized RCC, and is occasionally the presenting feature that prompts diagnostic evaluation [5]. When hypercalcemia occurs in advanced prostate cancer, it typically indicates osteolytic lesions or ectopic PTHrP production, both of which are markers of particularly aggressive disease biology [6]. By comparison, the occurrence of hypercalcemia in PSCC is exceptionally rare [7,8], with documentation limited to sporadic case reports. The paucity of literature reflects both the rarity of PC itself and the infrequency of this paraneoplastic manifestation. Nevertheless, when hypercalcemia develops in the setting of PSCC, it universally indicates advanced disease and portends poor prognosis. Consequently, the clinical and pathophysiological mechanisms, therapeutic responses and prognostic implications of this entity require comprehensive characterization.

Hypercalcemia represents a significant paraneoplastic complication, with an estimated incidence of 20–30% among cancer patients during an evolving disease. Malignancy-associated acute severe hypercalcemia represents an oncologic emergency requiring prompt recognition and interdisciplinary management [9,10]. Without timely recognition and intervention, this complication is life-threatening and is associated with substantial mortality rates, reaching 50% within 30 days of diagnosis [11,12,13].

The aim of this study was to systematically conduct a narrative review of the available evidence regarding tumor-induced hypercalcemia in PSCC and to synthesize existing evidence on clinical presentation, pathophysiological mechanisms, therapeutic strategies and outcomes. This could provide a structured framework to guide future clinical research.

## 2. Materials and Methods

**Figure 1:**
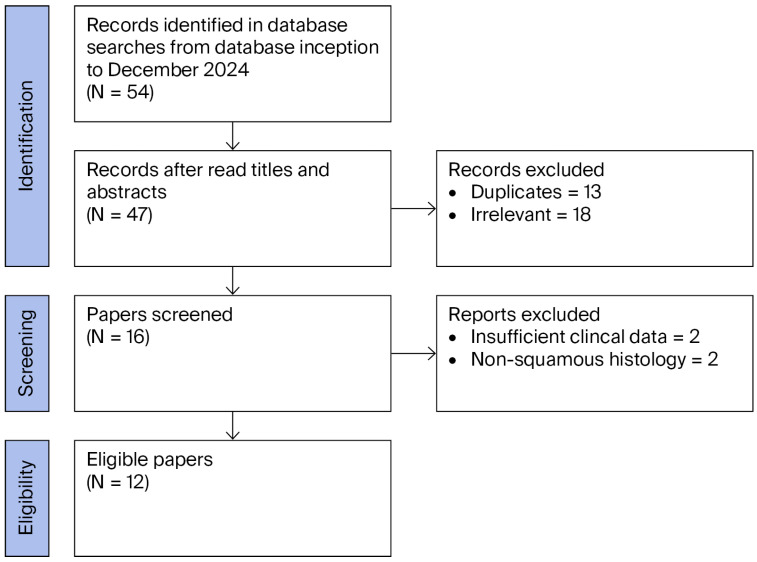
Flowchart of paper selection process.

A systematic literature search was conducted and reported in accordance with the PRISMA 2020 guidelines, across PubMed/MEDLINE and Scopus databases from database inception to December 2024. The following search terms were used without language filters: “penile cancer”, “squamous cell carcinoma,” “hypercalcemia,” and “paraneoplastic syndrome”. Supplementary manual searches were conducted for the bibliographies of all included articles to identify additional relevant studies (backward citation searching). To address potential gaps in primary search, supplementary screening was undertaken in ClinicalTrials.gov for any registered interventional or observational studies and in Google Scholar for additional case reports or gray literature. No registered clinical trials were identified, and no additional cases meeting all the inclusion criteria were found. A completed PRISMA 2020 checklist is provided in Supplementary Material S1. The PRISMA flow diagram documenting the study selection process is presented in Figure 1. This systematically conducted narrative review was registered retrospectively on the Open Science Framework (OSF) (registration DOI: 10.17605/OSF.IO/YB7NE).

Inclusion criteria: Histopathologically confirmed PSCC; biochemically documented hypercalcemia (serum calcium > 10.5 mg/dL or ionized calcium > 1.3 mmol/L); objective evidence of paraneoplastic etiology (elevated PTHrP, bone metastases, or exclusion of alternative causes); and published case reports, case series, or cohort studies with no language restrictions.

Exclusion criteria: Primary hyperparathyroidism or other non-malignant causes of hypercalcemia; insufficient clinical data to confirm diagnosis; and duplicate or irrelevant publications.

### Data Extraction

Data extraction was performed independently by two reviewers using a standardized extraction form. Extracted variables included: (1) patient demographics; (2) tumor-specific variables (histological grade, clinical stage, presence and anatomical distribution of metastases); (3) biochemical parameters (serum calcium concentrations, corrected calcium values, PTHrP levels, serum PTH, and alkaline phosphatase); (4) established etiology of hypercalcemia; (5) therapeutic strategies employed and (6) clinical outcomes (biochemical response, symptomatic improvement, and survival data).

Due to the rarity of the condition and heterogeneity of reports, data were synthesized descriptively using the median and range for continuous variables, frequencies and proportions for categorical variables, narrative synthesis of mechanistic pathways, and presentation of characteristics for individual cases.

## 3. Results

This systematically conducted narrative review identified 12 documented cases of hypercalcemia associated with PSCC published over a period of six decades across the medical literature, between 1965 and 2024 [14]. The key characteristics of the studies are summarized in Table 1.

### 3.1. Clinical and Tumor Characteristics

The median age at presentation was 56 years (with patients ranging between 45 and 83 years of age). Most patients had advanced disease, with 91.6% (11/12) presenting either local extension of the primary tumor (at least T3) or systemic development, at the time of hypercalcemia diagnosis [7,13,15,17,18,19,21,22,23,24,25,26]. Five cases presented with primary tumor stages T1/T2, but complicated by hypercalcemia, as reported by Anderson and Glenn in 1965 [7].

Constitutional symptoms associated with hypercalcemia were varied, including anorexia, fatigue or fever in the context of tumor-associated infection. Neurological symptoms occurred in seven cases and included altered mental status, confusion, somnolence, and lethargy [13,19,21,23,26]. Belrhali et al. specifically documented bradylalia (slow speech) as a manifestation of severe hypercalcemia in their report [24].

Gastrointestinal symptoms were reported in four cases, with dysphagia documented as a rare, but notable manifestation. Ayyathurai et al. described dysphagia, which improved concurrently with calcium normalization and recurred when calcium levels increased, suggesting a causal relationship [21]. Acute kidney injury (AKI) was reported in four cases, with serum creatinine elevations ranging from 1.7 to 6.25 mg/dL [13,15,21,26].

All 12 cases involved squamous cell carcinoma histology, but the tumor grade was specified in only 8 cases. Five of these cases presented with moderately differentiated (G2) tumors (five out of eight cases) [13,25,26]. In addition, well-differentiated (G1) tumors were identified in two cases [7,22], and a poorly differentiated (G3) tumor was observed in one case [15].

Primary tumor location was predominantly the penile glans penis (75%). Nodal tumor involvement was reported in all cases, with extracapsular extension (ECE) specifically described in four cases [15,25,26]. The presence of lymphovascular invasion (LVI) was noted in three cases [15,25,26].

### 3.2. Hypercalcemia Characteristics

Serum calcium levels at presentation had a median value of 15.45 mg/dL (12.6–18.6 mg/dL/3.14–4.64 mmol/L). Severe hypercalcemia (≥14 mg/dL) was documented in 66.7% of cases [13,15,21,23,26], and the highest documented level of 18.6 mg/dL was reported by Ho et al. [20]. Moderate hypercalcemia (12.6–14 mg/dL) occurred in four cases [7,15,18,25].

Distant metastases were documented in eight cases at the time of hypercalcemia diagnosis. The pattern of metastatic spread included: retroperitoneal lymph node metastases (two cases) [23,25], pulmonary metastases (four cases) [13,19,21,26], and bone metastases (two cases) [19,23]. The presence of visceral metastases was strongly associated with poor outcomes.

### 3.3. Mechanistic Classification

Published cases were stratified by the identified or inferred mechanism of hypercalcemia (Table 2). Two established pathophysiological mechanisms were identified: PTHrP-mediated humoral hypercalcemia and bone metastasis-associated hypercalcemia. In three cases, the mechanism remained undetermined due to the absence of PTHrP measurement. Given the small number of cases within each mechanistic subgroup (*n* = 3–6), these observations are descriptive and should be interpreted with caution. No definitive prognostic inferences could be drawn from subgroup comparisons. As such, these findings serve primarily as hypothesis-generating data for future studies.

#### 3.3.1. PTHrP-Mediated Humoral Hypercalcemia

Malignant humoral hypercalcemia is predominantly mediated by PTHrP, which accounts for approximately 80% of cases. Less frequent mechanisms include ectopic PTH secretion and increased 1,25-dihydroxyvitamin D production, typically associated with neuroendocrine and lymphoproliferative tumors [20,27]. PTHrP increases serum calcium through bone resorption but has limited effects on renal 1,25-dihydroxyvitamin D synthesis and intestinal calcium absorption [28]. Its biological action is mainly driven by stimulation of osteoclastic activity, which induces osteoblast expression of RANKL and suppresses osteoprotegerin, promoting osteoclast differentiation and activation [29]. It should be mentioned that 1,25-dihydroxyvitamin D-mediated hypercalcemia was not observed in any of the 12 reviewed cases. This is consistent with the established pathophysiology, as extrarenal production of 1,25-dihydroxyvitamin D is predominantly associated with lymphoproliferative malignancies (particularly Hodgkin and non-Hodgkin lymphoma) and granulomatous diseases, rather than squamous cell carcinomas, which characteristically produce PTHrP as the primary humoral mediator [30,31].

Malignant humoral hypercalcemia can be sustained by a biochemical profile of elevated PTHrP and suppressed PTH [23]. PTHrP elevation was documented in six cases [13,17,19,23,24,25], with levels ranging from 1.6 to 127 pmol/L (the reference range is typically <1.3–1.5 pmol/L). The highest PTHrP level was reported by Trejo-Rosales et al. at 127 pg/mL [13]. Concurrent PTH measurement was documented in five cases [13,17,22,23,24], highlighting suppressed levels in all instances, thus confirming the non-parathyroid-mediated mechanism of hypercalcemia.

Immunohistochemical confirmation of PTHrP production by tumor cells was described by Akashi et al.; specifically, strong brown immunohistochemical staining was obtained for PTHrP in PC tissue [19].

#### 3.3.2. Bone Metastasis-Associated Hypercalcemia

Bone scan metastases associated with hypercalcemia were documented in three cases [19,20,23]. Bone involvement included lytic lesions of the pelvis, vertebrae and ribs. It is worth noting that in the case reported by Ho et al., pathological fracture of the humerus represented the clinical manifestation of metastatic disease [20].

#### 3.3.3. Mechanism Undetermined (PTHrP Not Measured)

In three cases, hypercalcemia occurred in the absence of documented bone metastases and instead, in the context of large primary tumors or bulky nodal disease [7,18,26]. PTHrP levels were not measured in any of these cases, precluding definitive mechanistic classification. These cases are therefore classified as “mechanism undetermined.”

The observation that calcium normalization followed surgical resection of the primary tumor in selected cases [7,18] raises the hypothesis that bulky disease without bone metastases may involve an unmeasured humoral factor, most likely PTHrP, which drives the hypercalcemia. However, in the absence of PTHrP measurement, this remains speculative and cannot be presented as a confirmed mechanistic pathway. This category therefore represents a provisional classification based on available clinical data, rather than a confirmed distinct pathophysiological pathway.

### 3.4. Medical Management of Hypercalcemia

#### 3.4.1. Intravenous Hydration and Diuretics

Aggressive intravenous hydration with normal saline was used in all cases as an initial form of management (2500 to 8000 mL per 24 h). Loop diuretics (furosemide) were administered in 10 cases, with doses ranging from 40 to 120 mg daily. Despite being regarded as a first-line treatment [32], the response was limited in all cases. The calcium levels either failed to normalize or demonstrated only temporary improvement lasting mostly 24–72 h [7,13,15,16,17,18,19,20,21,22,23,24,26].

#### 3.4.2. Bisphosphonate Therapy

Bisphosphonates, such as pamidronate [19,21,24], etidronate [16], and zoledronic acid [13,20,22,23], were administered in 10 cases, representing the most used specific calcium-lowering therapy [33]. The response was documented, with calcium normalization achieved in six cases and a significant reduction observed in another two cases. The benefits were represented by the relatively fast impact (time to response—2 to 7 days) and the sustained effect (2–6 weeks).

#### 3.4.3. Corticosteroids

Corticosteroid therapy (dexamethasone or prednisone) can be used either alone or in combination with other molecules [34,35]. The response was described as variable, with one case showing no benefit [22] and in another case reporting modest calcium reduction [17].

#### 3.4.4. Tumor-Targeting Therapies

Tumor mediators most often originate in tumor tissue; this is true for the primary tumor, pathological lymph nodes, or secondary distant metastases. Even though favorable effects were reported in normalizing calcium levels when treating the primary tumor [7], surgery is described as having a significant impact, especially in cases with large lymph node metastases.

Another way in which we can act directly on tumor tissue, to indirectly control hypercalcemia, is radiotherapy. Although applied in several cases in the literature, the impact on mitigating calcemia was only described by Malakoff and Schmidt. They noted that when therapy was directed at metastases (external irradiation and bleomycin), sustained remission of hypercalcemia was observed, supporting the previously mentioned hypothesis [15].

It should be noted that these results are drawn from heterogeneous case reports spanning six decades with non-standardized treatment protocols.

### 3.5. Survival Outcome

Hypercalcemia is a life-threatening emergency, due to its unfavorable prognosis at the time of diagnosis. The median survival of reported cases is 9 weeks. The data varies from a 7 months survival, reported by Videtic et al., to a survival of 9 days, reported by Belrhali et al., in an 82-year-old patient with refractory hypercalcemia despite bisphosphonate therapy [16,24].

Although the general clinical picture includes neurological disorders and altered general condition at the time of presentation in most cases, the direct cause of mortality is not specified, except in the case presented by Sekita et al. The death of the 57-year-old patient was due to renal and respiratory failure, which are consequences of hypercalcemia [18].

The factors with considerable impact on survival among these patients include progressive hypercalcemia (>17 mg/dL), response to combined treatment and the presence of metastases [13,23,24]. In addition, Ayyathurai et al. noted that PTHrP-related hypercalcemia seems to be a strong indicator of reduced life expectancy for PC, as for other neoplasms [21].

### 3.6. Temporal Trends in Diagnosis and Management

Cases were categorized by decades of publication to identify the results of changes made to therapeutic and diagnostic strategies, due to an increased level of knowledge and experience. Between 1965 and 1989 (*n* = 4 cases), management was affected by limited biochemical characterization, the unavailability of PTHrP measurements, the lack of bisphosphonates, and the fact that basic therapy was only conducted as a form of supportive care. During the following period, from 1990 to 2009 (*n* = 4 cases), the PTHrP measurement became available, and bisphosphonate therapy emerged, improving mechanistic understanding. In recent times, from 2010 to 2024 (*n* = 4 cases), the routine PTHrP measurement has been identified alongside, standardized bisphosphonate protocols. Denosumab has also emerged as an alternative treatment and better supportive care is available to patients.

## 4. Discussion

This systematically conducted narrative review identified only 12 published cases of paraneoplastic hypercalcemia in PSCC over six decades, underscoring both the extreme rarity of this metabolic disorder and its clinical significance. The paucity of research in literature highlights both the uncommon nature of PSCC itself and the infrequency with which this paraneoplastic syndrome manifests even in advanced disease. The evidence base consists exclusively of case reports and small case series, limiting the ability to perform meta-analysis or establish definitive treatment algorithms. The present review synthesizes these reports to illustrate the spectrum of clinical presentations underlying mechanisms and therapeutic approaches described in this uncommon paraneoplastic syndrome.

Malignant humoral hypercalcemia mediated by PTHrP represents the most frequently reported pathway. Seven reports provide evidence of PTHrP involvement, with only six reporting elevated serum PTHrP levels and one (Sardiñas et al.) indicating borderline serum values, all in the setting of suppressed iPTH [13,18,19,21,22,23,24]. The immunohistochemical detection of PTHrP from tumor cells was documented by Akashi et al. and Sardinas et al., providing definitive evidence that ectopic hormone secretion as the causative mechanism [19,22].

The relationship between bone metastases and hypercalcemia in PSCC appears less common than the purely humoral mechanism [36]. Published cases describing osteolytic hypercalcemia emphasize the contribution of diffuse bone metastases, with some authors suggesting a mixed mechanism involving both direct bone destruction and humoral factors. A similar case is reported by Ho et al., without a PTHrP dosage. This particular case was diagnosed due to the presence of hypercalcemia, secondary to a pathological bone fracture [20].

The literature also indicates that surgical cytoreduction (debulking) may influence the development of calcium homeostasis in selected patients. This hypothesis is supported by Anderson and Glenn, who described the spontaneous normalization of calcium values following surgical treatment of the primary tumor [7]. In contrast, Malakoff and Schmidt reported a case, where primary tumor ablation had minimal effect on hypercalcemia, but subsequent therapy directed at metastatic lesions achieved calcium control. These differences suggest different anatomical tumor sites of hormonal discharge, which vary among patients [15].

Notably, the degree of hypercalcemia does not implicitly correlate with tumor volume or staging. Moderate biochemical changes have been described in patients with extensive loco-regional or metastatic disease, whereas severe hypercalcemia has also been reported with the absence of significant bone involvement. This observation reinforces the multifactorial nature of hypercalcemia in PSCC.

Clinical manifestations reported across published cases are complex, highlighting neurological symptoms (altered mental status, somnolence, and bradylalia), gastrointestinal dynamic disorders (loss of appetite and dysphagia), renal dysfunction and constitutional manifestations (weight loss and anorexia). Dysphagia is a rarely documented manifestation of hypercalcemia, attributed to reduced gastrointestinal smooth muscle contractility. Ayyathurai et al. reported it as a prominent symptom of the clinical picture [21].

Diagnostic evaluation requires the systematic exclusion of alternative etiologies of hypercalcemia and characterization of the underlying mechanism. This should include measurements of intact PTH, lactate dehydrogenase (LDH) and alkaline phosphatase (AP), followed by a PTHrP assay when initial observations suggest a paraneoplastic etiology [37].

The biochemical profile characteristic of PTHrP-mediated hypercalcemia implies suppressed PTH, hypophosphatemia, and absence of significantly elevated values of AP, in the absence of bone metastases. The confirmation of PTHrP elevation, when available, provides definitive evidence of the humoral mechanism. Immunohistochemical detection of PTHrP is rarely performed in clinical practice, but represents the gold standard for establishing ectopic hormone production, as performed by Akashi et al. [19].

Based on the evidence synthesized in this review and established principles of malignancy-associated hypercalcemia management, a structured diagnostic approach for suspected paraneoplastic hypercalcemia in PSCC can be proposed. The initial step should include confirmation of true hypercalcemia through measurements of albumin-corrected serum calcium or ionized calcium, followed by a biochemical panel comprising intact PTH, serum phosphate, alkaline phosphatase, serum creatinine, and lactate dehydrogenase. Suppressed intact PTH in the setting of elevated calcium strongly suggests a non-parathyroid-mediated mechanism and should prompt second-tier investigations including PTHrP assay and 1,25-dihydroxyvitamin D measurement. When PTHrP is elevated and has suppressed PTH, malignant humoral hypercalcemia can be confirmed. Imaging of stages at the time of diagnosis, including computed tomography of the chest, abdomen and pelvis, as well as bone scintigraphy, is a critical component of the evaluation [38,39]. Published cases consistently highlight the importance of comprehensive imaging at diagnosis to guide both metabolic and oncological management. In equivocal cases or when PTHrP is unavailable, immunohistochemical staining for PTHrP on tumor tissue should be considered, as demonstrated by Akashi et al. and Sardiñas et al. [19,22]. This systematic approach enables accurate mechanistic classification and guides the selection of targeted therapeutic strategies.

Management strategies described in the literature reflect the complexity of malignancy-associated hypercalcemia. Treatments have evolved considerably over the six decades spanned by the reviewed cases. The choice between bisphosphonates and denosumab represents an evolving area in the management of malignancy-associated hypercalcemia, but this depends on institutional resources.

Bisphosphonates have historically represented the cornerstone of specific calcium-lowering therapy. Zoledronic acid, the most potent intravenous bisphosphonate, achieves calcium normalization in 80–90% of patients with malignancy-associated hypercalcemia, with a median time to response of 4 days [40]. In the reviewed PSCC cases, bisphosphonate therapy achieved calcium normalization or a significant reduction in 8 of 10 treated patients, which is consistent with response rates reported in other types of squamous cell carcinomas [41,42]. However, the response was limited in time (2–6 weeks), necessitating repeated dosing or alternative strategies for sustained control.

While bisphosphonates have historically represented first-line therapy, denosumab, a fully human monoclonal antibody targeting RANKL, has demonstrated superior efficacy in some studies, particularly in cases with refractory bisphosphonate [43]. The pivotal study by Hu et al. demonstrated that denosumab achieved calcium normalization in 64% of patients who failed prior bisphosphonate therapy, with a median duration of 104 days for effective calcium control [43]. Its mechanism of action provides a distinct pharmacological approach, bypassing bisphosphonate resistance. Among the reviewed PSCC cases, Sardiñas et al. reported that the use of denosumab in combination with cinacalcet achieved biochemical control in a patient with borderline PTHrP elevation [22]. Kanta et al. similarly used denosumab following initial zoledronic acid therapy [23]. These observations suggest that denosumab may be an efficient alternative in bisphosphonate-refractory PSCC-associated hypercalcemia, consistent with evidence from other solid tumors [44,45]. These approaches align with contemporary management principles for severe malignant hypercalcemia [22,23].

Calcitonin, although not extensively documented in the reviewed PSCC cases, is an adjunctive agent to consider in the acute setting. Its rapid onset of action (within 4–6 h) provides a bridge to the delayed effect of bisphosphonates (48–96 h) and it can be particularly useful in patients with severe symptomatic hypercalcemia requiring immediate calcium reduction [34]. An emerging consideration in the management of PC involves the increasing use of immune checkpoint inhibitors for documented cases of hypercalcemia occurring despite combined anti-PD-L1 and anti-EGFR therapy [46]. This iatrogenic etiology distinction is clinically important, as immunotherapy-related hypercalcemia may require different management strategies, including immunosuppressive therapy and standard calcium-lowering measures.

The occurrence of paraneoplastic hypercalcemia in PSCC uniformly indicates a poor prognosis. The survival outcome following hypercalcemia diagnosis ranges from 9 days, as reported by Belrhali et al. [24], with rapid clinical deterioration after admission, despite pamidronate, to 7 months reported by Videtic et al. [16].

The prognostic significance of hypercalcemia likely reflects the advanced tumor biology, rather than the metabolic derangement itself. While aggressive management can achieve biochemical correction, this intervention rarely translates into meaningful survival prolongation, emphasizing the need for early detection and treatment of PSCC before the development of such complications [38].

Systematic reporting and collaborative data collection are needed to improve understanding of pathophysiology, optimize management strategies and refine prognostic assessment.

### 4.1. Comparison with Other Squamous Cell Carcinomas

Paraneoplastic hypercalcemia in PSCC shares the fundamental characteristics observed in the SCC of other anatomical sites, particularly those of the head and neck (HNSCC) and lung (LSCC). PTHrP-mediated humoral hypercalcemia is predominant, and its clinical presentation is similar. The bisphosphonate response rates are comparable (75–85%) and hypercalcemia universally signals advanced disease biology with poor prognosis [3,34,42,43,44,47,48,49,50]. These parallels confirm that the propensity for PTHrP production and subsequent hypercalcemia represents a characteristic of squamous cell differentiation rather than a site-specific phenomenon. The molecular basis for this shared predisposition involves upregulation of PTHrP gene expression through the EGFR signaling cascade, TGF-β pathway and Ras-MAPK signaling, with the transcription factors constitutively activated in SCC acting as drivers of ectopic PTHrP production across anatomical sites [51,52].

One clinically relevant difference between PSCC and other SCC types is the potential for surgical cytoreduction. While debulking surgery is often limited in advanced HNSCC due to anatomical complexity, PSCC may offer greater surgical accessibility for both primary tumor and inguinal lymph node debulking. As such, calcium normalization followed surgical procedures in selected PSCC cases suggests that cytoreductive surgery may have a more prominent role in the multimodal management of PSCC-associated hypercalcemia compared to other SCC sites [7,26]. This represents a potential unique advantage in the treatment of PSCC that warrants further investigation. Additionally, as immune checkpoint inhibitors become increasingly integrated into the treatment of advanced PC, clinicians should be aware of immunotherapy-related hypercalcemia as a distinct differential diagnosis [46].

### 4.2. Study Limitations

The systematically conducted narrative review has several inherent limitations. First, the retrospective nature of the study collection limits generalizability and precludes meta-analysis. Publication bias exists, with the evidence base consisting of uncontrolled, retrospective severe case reports, which carry a substantial risk of bias. Case reports are subject to selective reporting, with a tendency to document and publish clinically dramatic presentations (severe hypercalcemia or unusual manifestations) rather than milder or successfully managed cases. This introduces an ascertainment bias that likely overestimates the severity of hypercalcemia at presentation and the linearity of poor prognosis observed. Furthermore, the absence of control groups precludes any causal inference regarding the relationship between specific therapeutic interventions and outcomes. Moreover, the 60-year timespan of the included cases introduces substantial temporal heterogeneity. Diagnostics, treatment protocols, and reporting standards evolved considerably over this period. Therapeutic responses observed in earlier cases managed with supportive care alone cannot be meaningfully compared with contemporary treatments and this temporal confounding further limits the interpretability of treatment-related observations.

Second, the measurement of PTHrP was inconsistently performed across published cases (in only 58% of cases) and management approaches varied substantially. The lack of standardization of diagnostic and treatment protocols among the cases reported to date limits a direct comparison of outcomes. This heterogeneity limits the ability to definitively categorize pathophysiological mechanisms in these cases. In three cases where PTHrP was not measured, mechanistic classification could not be established, and these were accordingly categorized as “mechanism undetermined.” The absence of PTHrP measurement in about 42% of cases represents a fundamental limitation that precludes comprehensive mechanistic characterization of the entire cohort.

Third, the rarity of this metabolic disorder limits statistical analysis and the conclusions drawn should only be considered advisory and hypothesis-generating rather than definitive. The survival outcomes were incompletely reported in several cases, with various follow-up duration and inconsistent documentation of the cause of death.

Finally, this review was limited to the published literature indexed in PubMed/MEDLINE and Scopus, as well as supplementary screening of ClinicalTrials.gov and Google Scholar. Additional cases may exist in non-indexed regional journals or unpublished institutional records. A truly comprehensive understanding of this rare entity would require international collaboration between registries.

Despite these limitations, this review provides the most comprehensive synthesis to date of paraneoplastic hypercalcemia in PSCC. These findings should be interpreted within the constraints of the available evidence, and all conclusions should be regarded as hypothesis-generating. Nevertheless, this synthesis provides a structured framework for the clinical recognition and management of this rare entity, establishing a foundation for future standardized case reports.

### 4.3. Future Research Perspectives

Several critical knowledge gaps require future research. In terms of molecular status, genomic profiling of PTHrP-secreting PSCC is necessary to identify the drivers of ectopic production. Investigations should also be conducted on the regulation of PTHrP promoters and the correlation of PTHrP expression patterns with tumor characteristics. Regarding the role of PTHrP, an investigation of this biomarker as a surrogate endpoint in clinical trials is needed to develop predictive models that incorporate PTHrP with staging parameters.

In cases presenting hypercalcemia in the setting of bulky disease, without bone metastases or available PTHrP data, the underlying mechanism remains undetermined. Future cases should include a mandatory PTHrP measurement to clarify whether these represent scenarios of unmeasured humoral hypercalcemia or whether an alternative mechanism exists. Regarding the potential utility of PTHrP as a biomarker, prospective studies are needed to evaluate whether serial PTHrP monitoring can serve as a marker of treatment response and disease recurrence. Predictive models that correlate PTHrP levels with already known staging parameters should be developed to refine the prognostic outcomes in advanced PSCC.

From a therapeutic perspective, the optimal sequencing of calcium-lowering agents in PSCC-associated hypercalcemia should be refined. Direct comparisons of bisphosphonates versus denosumab in SCC-associated hypercalcemia would provide valuable guidance applicable to PSCC. This analysis would most likely be performed retrospectively on large cohorts of patients, as prospective studies on life-threatening pathologies at the time of presentation are difficult to implement.

Finally, the extreme rarity of this entity underscores the need for international collaborative efforts. The establishment of a multicenter registry could be modeled on existing rare-tumor databases and integrated with ongoing PC collaborative networks. Clinical practice could be optimized through the development of evidence-based clinical practice guidelines and QoL studies comparing aggressive versus conservative management.

## 5. Conclusions

Paraneoplastic hypercalcemia represents a rare but clinically significant complication of advanced PSCC. The predominant pathophysiological mechanism is PTHrP-mediated humoral hypercalcemia, with bone metastasis-associated osteolysis representing an additional pathway. In a subset of cases, the mechanism could not be determined due to the absence of PTHrP measurement. Management strategies range from medical therapy with bisphosphonates or denosumab to surgical cytoreduction, with individualized approaches based on tumor characteristics and patient factors. Despite aggressive supportive care and metabolic correction, the prognosis remains poor, with hypercalcemia serving as a marker of advanced disease biology. Standardized reporting of future cases with mandatory PTHrP measurement and international registry collaboration is essential to advance the understanding of this rare, but life-threatening paraneoplastic entity.

## Figures and Tables

**Table 1:** Summary of published cases of hypercalcemia associated with PSCC.

Author, Year	Age (yr)	Clinical Stage	Ca (mg/dL)	PTHrP	Bone Mets	Treatment	Outcome *
Anderson and Glenn, 1965 [7]	60	T2N3	12.6	ND	No	Surgery (primary tumor)	No survival data
Malakoff and Schmidt, 1975 [15]	53	T2N3	16.0	ND	No	Mithramycin + surgery + RT + CHT	Died after 3 mo
Videtic et al., 1997 [16]	53	T2N3M1	13.0	ND	No	CHT + RT + bisphosphonate (etidronate)	Died after 7 mo
Dorfinger et al., 1999 [17]	42	T2N3M0	13.71	ND	No	Bisphosphonate + surgery (tumor debulking)	Died after 21 days
Sekita et al., 1999 [18]	57	rT3N3	18	695 pmol/L (13.8–55.3)	No	Bisphosphonate	Died after 2 wk
Akashi et al., 2002 [19]	53	T3N2M1	15.5	4.0 pmol/L (normal < 1.1)	Yes	CHT + bisphosphonate (pamidronate)	Died after 4 mo
Ho et al., 2006 [20]	55	T3N2M1	18.6	ND	Yes (fracture)	Bisphosphonate (zoledronic acid)	No survival data
Ayyathurai et al., 2007 [21]	75	T2N2M0	15.1	6.5 pmol/L	No	Bisphosphonate (pamidronate) + RT	Died after 7 wk
Trejo-Rosales et al., 2014 [13]	47	T4N3M1	16.9	13.46 pmol/L	No	Bisphosphonate (zoledronic acid)	Died (timing ND)
Sardiñas et al., 2018 [22]	48	T3N3M0	13.4	1.9 pmol/L	No	Bisphosphonate (ZA) + cinacalcet + denosumab + RT	Died after 2 mo
Kanta et al., 2020 [23]	45	T2N3M1	17.0	120 pmol/L	Yes	Bisphosphonate (ZA) + denosumab	Died after 24 days
Belrhali et al., 2024 [24]	82	T3N3M1	15.6	3.88 pmol/L	No	Bisphosphonate (pamidronate)	Died after 9 days

* Outcomes calculated from the time of hypercalcemia diagnosis. Abbreviations: Ca, serum calcium; PTHrP, parathyroid hormone-related peptide; Mets, metastases; ND, not documented; RT, radiation therapy; CHT, chemotherapy; ZA, zoledronic acid; wk, weeks; mo, months; yr, years.

**Table 2:** Biochemical characteristics of hypercalcemia in PSCC by mechanistic classification.

Pathophysiological Mechanism	Cases (*n*)	Median Serum Calcium, mg/dL (Range)	PTHrP Status	PTH Status	Bone Metastases
PTHrP-mediated humoral hypercalcemia	6	15.8 (13.2–18.6)	Elevated (1.6–127 pmol/L)	Suppressed	28.6% (2/7)
Bone metastasis-associated hypercalcemia	3	16.2 (14.8–18.6)	Variable (measured in 1/3)	Suppressed	100% (3/3)
Mechanism undetermined (PTHrP not measured) ^†^	3	14.1 (12.6–15.0)	Not measured	Not documented	0% (0/3)

^†^ In three cases, PTHrP was not measured, and mechanistic classification could not be determined. The absence of bone metastases and the presence of bulky disease raise the hypothesis of an unmeasured humoral factor (likely PTHrP), but this remains speculative. Abbreviations: PTHrP, parathyroid hormone-related peptide; PTH, parathyroid hormone.

## Data Availability

The original contributions presented in this study are included in the article. Further inquiries can be directed to the corresponding author.

## Supplementary Materials

The following supporting information can be downloaded at: https://www.mdpi.com/article/10.3390/jcm15051809/s1, Supplementary Material S1: PRISMA 2020 checklist.

## Abbreviations

The following abbreviations are used in this manuscript:

PSCC	Penile squamous cell carcinoma
PC	Penile cancer
PTH	Parathyroid hormone
PTHrP	Parathyroid hormone-related peptide
AKI	Acute kidney injury
RANKL	Receptor activator of nuclear factor kappa-B ligand
RT	Radiation therapy
CHT	Chemotherapy
ECE	Extracapsular extension
LVI	Lymphovascular invasion
LDH	Lactate dehydrogenase
AP	Alkaline phosphatase
LS	Lichen sclerosus
SCC	Squamous cell carcinoma
HNSCC	Head and neck squamous cell carcinoma
LSCC	Lung squamous cell carcinoma
